# Supplementary material for: Empirical methods for controlling false positives and estimating confidence in ChIP-Seq peaks
Source: BMC Bioinformatics. 2008 Dec 5;9:523. doi: 10.1186/1471-2105-9-523 (PMC2628906; doi:10.1186/1471-2105-9-523)
Supplement: Additional File 1 — A variety of html documents from the USeq web site detailing the available applications, their best usage, output file type descriptions, command line menus, etc. [file 1471-2105-9-523-S1.zip › USeqUserGuides/JavaDoc.zip!/JavaDoc/trans/main/Interval.html]

Interval


|  |  |  |
| --- | --- | --- |
| **Overview** \| **Package** \| **Class** \| **Use** \| **Tree** \| **Deprecated** \| **Index** \| **Help** | |  |
| **PREV CLASS**   **NEXT CLASS** | **FRAMES**    **NO FRAMES**     **All Classes** |  |
| SUMMARY: NESTED \| FIELD \| CONSTR \| METHOD | DETAIL: FIELD \| CONSTR \| METHOD |  |


---


## trans.main Class Interval

```
java.lang.Object
  trans.main.Interval
```

**All Implemented Interfaces:**: java.io.Serializable, java.lang.Comparable

---

``` public class Interval extends java.lang.Object implements java.lang.Comparable, java.io.Serializable ```

Container for holding information about a potential enriched region: windows, oligos, sub windows, binding peaks....

**See Also:**: Serialized Form

---

**Constructor Summary**

`Interval(Window window,
int sizeOfOligo)`


| **Method Summary** | |
| --- | --- |
| `int` | `compareTo(java.lang.Object obj)` |
| `Oligo[]` | `extractSubRegionOligos(int start, int end)`             Given a bp start and bp end as well as a larger Oligo[] returns a sub array of the oligos flanking and including the start and end. |
| `double[]` | `getBaseScores()` |
| `SubWindow` | `getBestSubWindow()` |
| `java.lang.String` | `getBestSubWindowSequence()` |
| `Window` | `getBestWindow()` |
| `java.lang.String` | `getBestWindowSequence()` |
| `BindingPeak[]` | `getBindingPeaks()` |
| `java.io.File[]` | `getCelFiles()` |
| `java.lang.String` | `getChromosome()` |
| `double[]` | `getFractionIntersections()` |
| `int` | `getMaxCluster()` |
| `double` | `getMotifHitSum()`             Sums the logged motif hit values using the best AID window or the entire interval. |
| `int` | `getNumberControlIntensities()` |
| `int` | `getNumberMotifHits()` |
| `int` | `getNumberOfWindows()` |
| `int` | `getNumberTreatmentIntensities()` |
| `Oligo[]` | `getOligos()` |
| `java.lang.String[]` | `getRegionNames()` |
| `java.lang.String` | `getSequence()` |
| `int` | `getSizeOfOligoMinusOne()` |
| `double` | `getSortBy()` |
| `int` | `getStart1stOligo()` |
| `int` | `getStartLastOligo()` |
| `boolean` | `isBestWindowScored()` |
| `void` | `setBaseScores(double[] baseScores)` |
| `void` | `setBestSubWindow(SubWindow bestSubWindow)` |
| `void` | `setBestWindow(Window bestWindow)` |
| `void` | `setBestWindowScored(boolean bestAIDWindowScored)` |
| `void` | `setBindingPeaks(BindingPeak[] bindingPeaks)` |
| `void` | `setCelFiles(java.io.File[] celFiles)` |
| `void` | `setChromosome(java.lang.String string)` |
| `void` | `setFractionIntersections(double[] percentIntersections)` |
| `void` | `setMaxCluster(int maxCluster)` |
| `void` | `setNumberControlIntensities(int numberControlIntensities)` |
| `void` | `setNumberMotifHits(int motifHits)` |
| `void` | `setNumberOfWindows(int numberOfWindows)` |
| `void` | `setNumberTreatmentIntensities(int numberTreatmentIntensities)` |
| `void` | `setOligos(Oligo[] oligos)` |
| `void` | `setRegionNames(java.lang.String[] repeatNames)` |
| `void` | `setSequence(java.lang.String sequence)` |
| `void` | `setSizeOfOligoMinusOne(int sizeOfOligoMinusOne)` |
| `void` | `setSortBy(double sortBy)` |
| `void` | `setStart1stOligo(int i)` |
| `void` | `setStartLastOligo(int i)` |
| `java.lang.String` | `toString()` |

**Methods inherited from class java.lang.Object**

`equals, getClass, hashCode, notify, notifyAll, wait, wait, wait`

**Constructor Detail**

### Interval

```
public Interval(Window window,
                int sizeOfOligo)
```


**Method Detail**

### compareTo

```
public int compareTo(java.lang.Object obj)
```

:   **Specified by:**: `compareTo` in interface `java.lang.Comparable`

---


### getMotifHitSum

```
public double getMotifHitSum()
```

:   Sums the logged motif hit values using the best AID window or the entire interval.
    This is equivalent to the product of the probabilities.

---


### toString

```
public java.lang.String toString()
```

:   **Overrides:**: `toString` in class `java.lang.Object`

---


### extractSubRegionOligos

```
public Oligo[] extractSubRegionOligos(int start,
                                      int end)
```

:   Given a bp start and bp end as well as a larger Oligo[] returns a sub array of the oligos flanking and including
    the start and end.

---


### getBestWindowSequence

```
public java.lang.String getBestWindowSequence()
```

---


### getBestSubWindowSequence

```
public java.lang.String getBestSubWindowSequence()
```

---


### getChromosome

```
public java.lang.String getChromosome()
```

---


### getStartLastOligo

```
public int getStartLastOligo()
```

---


### getStart1stOligo

```
public int getStart1stOligo()
```

---


### setChromosome

```
public void setChromosome(java.lang.String string)
```

---


### setStartLastOligo

```
public void setStartLastOligo(int i)
```

---


### setStart1stOligo

```
public void setStart1stOligo(int i)
```

---


### getNumberMotifHits

```
public int getNumberMotifHits()
```

---


### setNumberMotifHits

```
public void setNumberMotifHits(int motifHits)
```

---


### getSequence

```
public java.lang.String getSequence()
```

---


### setSequence

```
public void setSequence(java.lang.String sequence)
```

---


### getMaxCluster

```
public int getMaxCluster()
```

---


### setMaxCluster

```
public void setMaxCluster(int maxCluster)
```

---


### setSortBy

```
public void setSortBy(double sortBy)
```

---


### getSortBy

```
public double getSortBy()
```

---


### getNumberOfWindows

```
public int getNumberOfWindows()
```

---


### setNumberOfWindows

```
public void setNumberOfWindows(int numberOfWindows)
```

---


### getOligos

```
public Oligo[] getOligos()
```

---


### setOligos

```
public void setOligos(Oligo[] oligos)
```

---


### getBestWindow

```
public Window getBestWindow()
```

---


### setBestWindow

```
public void setBestWindow(Window bestWindow)
```

---


### getCelFiles

```
public java.io.File[] getCelFiles()
```

---


### setCelFiles

```
public void setCelFiles(java.io.File[] celFiles)
```

---


### getSizeOfOligoMinusOne

```
public int getSizeOfOligoMinusOne()
```

---


### getNumberControlIntensities

```
public int getNumberControlIntensities()
```

---


### setNumberControlIntensities

```
public void setNumberControlIntensities(int numberControlIntensities)
```

---


### getNumberTreatmentIntensities

```
public int getNumberTreatmentIntensities()
```

---


### setNumberTreatmentIntensities

```
public void setNumberTreatmentIntensities(int numberTreatmentIntensities)
```

---


### getBaseScores

```
public double[] getBaseScores()
```

---


### setBaseScores

```
public void setBaseScores(double[] baseScores)
```

---


### getBestSubWindow

```
public SubWindow getBestSubWindow()
```

---


### setBestSubWindow

```
public void setBestSubWindow(SubWindow bestSubWindow)
```

---


### isBestWindowScored

```
public boolean isBestWindowScored()
```

---


### setBestWindowScored

```
public void setBestWindowScored(boolean bestAIDWindowScored)
```

---


### getBindingPeaks

```
public BindingPeak[] getBindingPeaks()
```

---


### setBindingPeaks

```
public void setBindingPeaks(BindingPeak[] bindingPeaks)
```

---


### getFractionIntersections

```
public double[] getFractionIntersections()
```

---


### setFractionIntersections

```
public void setFractionIntersections(double[] percentIntersections)
```

---


### getRegionNames

```
public java.lang.String[] getRegionNames()
```

---


### setRegionNames

```
public void setRegionNames(java.lang.String[] repeatNames)
```

---


### setSizeOfOligoMinusOne

```
public void setSizeOfOligoMinusOne(int sizeOfOligoMinusOne)
```


---


|  |  |  |
| --- | --- | --- |
| **Overview** \| **Package** \| **Class** \| **Use** \| **Tree** \| **Deprecated** \| **Index** \| **Help** | |  |
| **PREV CLASS**   **NEXT CLASS** | **FRAMES**    **NO FRAMES**     **All Classes** |  |
| SUMMARY: NESTED \| FIELD \| CONSTR \| METHOD | DETAIL: FIELD \| CONSTR \| METHOD |  |


---
